# A Wavelet-Based Bilateral Segmentation Study for Nanowires

**DOI:** 10.3390/nano15211612

**Published:** 2025-10-23

**Authors:** Yuting Hou, Yu Zhang, Fengfeng Liang, Guangjie Liu

**Affiliations:** School of Computer Science and Technology, Changchun Normal University, Changchun 130032, China

**Keywords:** one-dimensional nanowires, semantic segmentation, deep learning, wavelet-based convolution, BiSeNetV1, feature extraction

## Abstract

One-dimensional (1D) nanowires represent a critical class of nanomaterials with extensive applications in biosensing, biomedicine, bioelectronics, and energy harvesting. In materials science, accurately extracting their morphological and structural features is essential for effective image segmentation. However, 1D nanowires frequently appear in dispersed or entangled configurations, often with blurred backgrounds and indistinct boundaries, which significantly complicates the segmentation process. Traditional threshold-based methods struggle to segment these structurally complex nanowires with high precision. To address this challenge, we propose a wavelet-based Bilateral Segmentation Network named WaveBiSeNet, to which a Dual Wavelet Convolution Module (DWCM) and a Flexible Upsampling Module (FUM) are introduced to enhance feature representation and improve segmentation accuracy. In this study, we benchmarked WaveBiSeNet against ten segmentation models on a peptide nanowire image dataset. Experimental results demonstrate that WaveBiSeNet achieves, mIoU of 77.59%, an accuracy of 89.95%, an F1 score of 87.22%, and a Kappa coefficient of 74.13%, respectively. Compared to other advanced models, our proposed model achieves better segmentation performance. These findings demonstrate that WaveBiSeNet is an end-to-end deep segmentation network capable of accurately analyzing complex 1D nanowire structures.

## 1. Introduction

One-dimensional (1D) nanowires [1] are linear nanostructures with cross-sectional dimensions typically ranging from 1 to 100 nanometers, in which the length is much greater than the diameter. Their lengths can extend from several hundred nanometers to several millimeters or even longer, making them a representative class of one-dimensional materials. Depending on their composition and application, 1D nanowires can be classified into several categories. Semiconductor nanowires (e.g., silicon, gallium nitride) are widely used in electronic and optoelectronic devices [2]; metal nanowires (e.g., copper, gold) exhibit outstanding thermal conductivity [3]; oxide nanowires (e.g., zinc oxide) demonstrate excellent performance in photocatalysis and gas sensing [4]; carbon-based nanowires (e.g., carbon nanotubes) combine high strength with superior electrical conductivity [5]; and peptide nanowires, due to their self-assembly capability and biocompatibility, are extensively employed in biomedical and biosensing applications [6,7]. In general, these nanowires possess remarkable electrical conductivity, mechanical flexibility, and tunable physicochemical properties, rendering them highly promising for applications in sensing technologies, energy devices, and biomedical engineering [8].

In materials science, the accurate measurement of morphological parameters of one-dimensional (1D) nanomaterials, such as length, diameter, curvature, and spatial distribution, is essential for understanding material performance, analyzing electrocatalytic activity, and optimizing the design of functional devices. Transmission electron microscopy (TEM) [9] is widely employed to characterize nanowire structures; however, it is inherently limited to providing localized structural information [10]. TEM images of one-dimensional nanowires often exhibit blurred backgrounds, overlapping structures, and adhesion effects, which complicate accurate boundary delineation. Traditional image processing methods, including edge detection, contrast enhancement, and thresholding, are highly sensitive to image quality and frequently rely on manual intervention, rendering them unsuitable for complex structural environments. While semi-automatic annotation tools such as LabelMe [11] offer some assistance, they suffer from limited edge-detection precision and low efficiency for large-scale image datasets. Compared with higher-dimensional objects, segmenting one-dimensional nanowires from the background remains a significant challenge in computer vision [12].

In recent years, deep learning has been widely applied in the field of nanomaterials due to its end-to-end training mechanism and excellent feature extraction capabilities [13,14]. Compared with conventional methods, deep learning offers greater accuracy and robustness in nanowire segmentation. It enables the automatic learning of optimal features, reduces human involvement, and improves generalization across complex image distributions.

This study proposes WaveBiSeNet, a wavelet-based bilateral segmentation network based on the BiSeNetV1 [15] architecture, for the high-precision segmentation of one-dimensional nanowires. The model incorporates a Dual Wavelet Convolution Module (DWCM) to enhance feature extraction and a Flexible Upsampling Module (FUM) to improve edge detail reconstruction. Experiments were conducted on a dataset of 3035 peptide nanowire TEM images, which include both dispersed and networked structures with complex backgrounds and blurred edges. Comparative evaluation against ten semantic segmentation models shows that WaveBiSeNet achieves 77.59% mIoU, 89.95% accuracy, 87.22% F1 score, and 74.13% Kappa, demonstrating accurate segmentation of 1D nanowires under challenging imaging conditions. We hypothesize that, under the same evaluation protocol, the integration of DWCM and FUM enables WaveBiSeNet to improve segmentation in blurred backgrounds and adherent regions compared with BiSeNetV1.

The contributions of this paper are summarized as follows:

(1) We propose WaveBiSeNet, a wavelet-based bilateral segmentation network, that improves upon BiSeNetV1 [15] for the accurate segmentation of one-dimensional nanowires with complex backgrounds and blurred edges.
(2) We introduce the Dual Wavelet Convolution Module (DWCM), which enhances feature extraction, and the Flexible Upsampling Module (FUM), which refines fine edge details.
(3) Experiments on the peptide nanowire dataset demonstrate that WaveBiSeNet outperforms ten existing semantic segmentation models.

## 2. Related Work

In the early stages, machine learning was applied to predict the properties of nanomaterials and assist in their design, promoting the development of computational approaches in nanomaterial research [16,17]. Several studies have demonstrated the utility of machine learning in this domain. Ghada Dahy et al. [18] proposed an intelligent optimization model capable of classifying different types of nanoparticles. Ajay Vikram Singh et al. [19] applied machine learning techniques to investigate interactions between cells and nanoparticles (NPs). Byoungsang Lee et al. [20] conducted high-precision statistical analysis of large-scale nanoparticle morphologies using machine learning methods. Arkaprava Banerjee et al. [21] effectively predicted the cytotoxicity of TiO_2_-based multi-component nanomaterials through hyperparameter optimization and structural similarity analysis. Parashuram Bannigidad et al. [22] utilized probabilistic neural networks (PNN) and k-nearest neighbor (K-NN) classifiers to distinguish between boron, iron, and silver nanoparticles. Although machine learning has accelerated research and development in nanomaterials, challenges remain, particularly in terms of data quality and algorithm interpretability [23].

With the advancement of deep learning, its applications in nanomaterials research have become more sophisticated. Recent studies increasingly focused on the image analysis and feature extraction of one-dimensional (1D) nanowire materials. Jacob R. Taylor et al. [24] employed a Vision Transformer [25] to predict Majorana zero modes (MZMs) in 1D superconducting-semiconductor nanowires. Junghyun Lee et al. [26] applied an artificial neural network (ANN) [27] to predict the electromagnetic response of double-layer nanowire gratings. Huitian Bai et al. [28] proposed a bidirectional long short-term memory (LSTM) model combined with a conditional random field layer for the precise localization and orientation detection of nanowires. Linmao Li et al. [29] integrated Mask R-CNN [30] and YOLO [31] to identify and segment nanowire structures in SEM images. Jack D. Kendall et al. [32] developed a neuromorphic network architecture (MN3) based on resistive nanowires, enabling efficient deep neural network training. Xin Hu et al. [33] used convolutional neural networks to perform the super-resolution reconstruction and deblurring of silver nanowires and other nanostructures in blurred optical images.

Despite these advancements, semantic segmentation of one-dimensional (1D) nanowires remains limited. This study proposes a segmentation framework tailored to the structural characteristics of 1D nanowire materials. By integrating deep learning-based semantic segmentation techniques, the framework improves segmentation accuracy and recognition performance for these structures.

## 3. Materials and Methods

This section presents the baseline BiSeNetV1 [15] and the improved WaveBiSeNet architecture. WaveBiSeNet incorporates two specialized modules: the Dual Wavelet Convolution Module (DWCM), which enhances feature extraction, and the Flexible Upsampling Module (FUM), which refines edge detail reconstruction. Together, these modules enable the accurate segmentation of one-dimensional nanowires, particularly in images with complex backgrounds and blurred edges.

### 3.1. BiSeNetV1 Model Architecture

**Figure 1:**
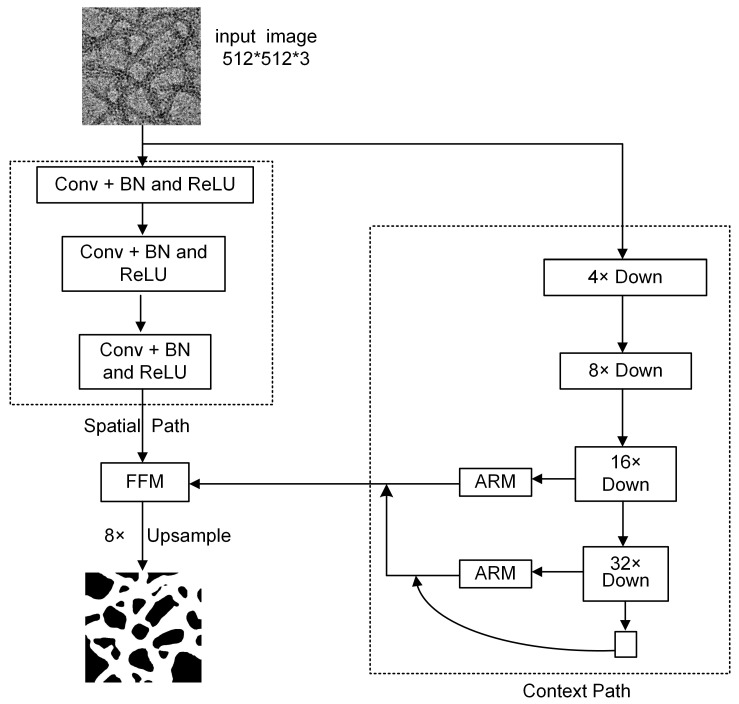
Structure of the BiSeNetV1 network.

**Figure 2:**
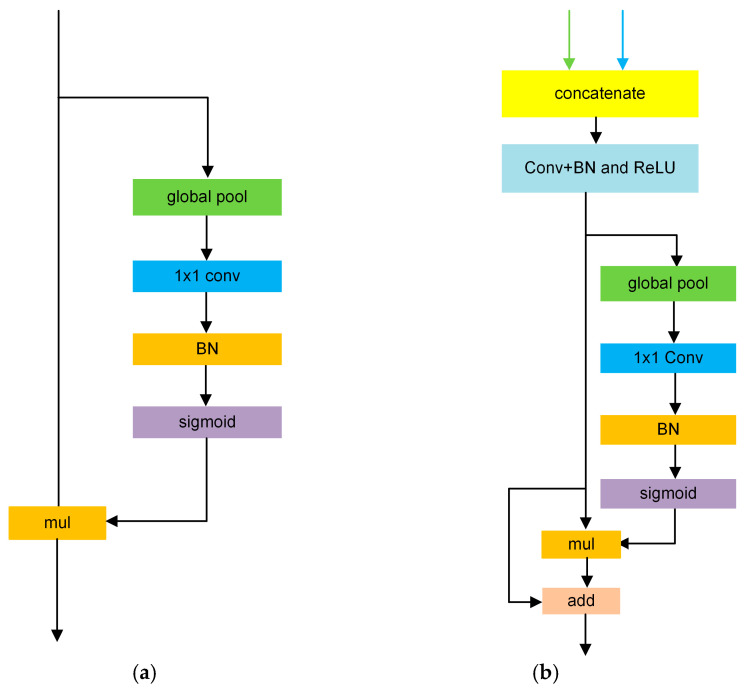
Structure of the Attention Refinement Module and the Feature Fusion Module. (**a**) ARM; (**b**) FFM.

The BiSeNetV1 [15] dual-branch real-time semantic segmentation network is designed to strike a balance between segmentation accuracy and computational efficiency. It comprises two parallel paths: a spatial path for preserving fine-grained details and a context path for capturing high-level semantic information. The overall network architecture is illustrated in Figure 1. In the spatial path, three convolutional layers with a kernel size of 3 × 3 and a stride of 2 are employed to progressively downsample the input image, ultimately reducing its resolution to 1/8 of the original size while retaining spatial structure information. In parallel, the contextual path utilizes ResNet-18 [34] as the backbone to extract deep semantic features. This path includes 8× and 16× downsampling operations and integrates a global average pooling module to enhance the model’s global context awareness. To further refine the extracted features, an Attention Refinement Module (ARM) is incorporated, which enhances critical features and suppresses irrelevant information. The structure of the ARM is shown in Figure 2a. Specifically, the ARM first applies global average pooling (GAP) to the input feature map to obtain channel-wise global response statistics. These statistics are then passed through a 1 × 1 convolution layer, followed by normalization and a Sigmoid activation function, to generate channel attention weights. Finally, these weights are multiplied channel-wise with the original feature map, thereby emphasizing task-relevant information while suppressing background noise and irrelevant responses. Furthermore, the features processed by ARM at the 1/16 scale are combined with the global contextual information obtained from the GAP branch and then upsampled to the 1/8 scale. These enriched features are fused with shallow features from earlier layers, effectively injecting global semantic information into higher-resolution representations.

The outputs from both paths are fused by a Feature Fusion Module (FFM). The structure of the FFM is shown in Figure 2b. FFM in BiSeNetV1 integrates the spatial and context pathways at the 1/8 scale. The two feature maps are concatenated along channels and passed through a 3 × 3 convolution, batch normalization (BN), and ReLU to perform local interaction and unify the channel dimensionality to a fixed size. A lightweight channel-attention branch applies global average pooling followed by a 1 × 1 convolution, batch normalization, and a sigmoid to obtain channel-wise coefficients ω ϵ (0, 1). These coefficients recalibrate the main stream via channel-wise multiplication, and a residual addition with the pre-attention feature yields the final output. This design injects high-level semantics while preserving fine-grained spatial details, effectively combining detailed spatial features with rich contextual semantics. Finally, the fused feature map is upsampled to the original resolution using 8× bilinear interpolation, enabling accurate, pixel-level semantic predictions.

### 3.2. The Structure of the Proposed WaveBiSeNet Model

**Figure 3:**
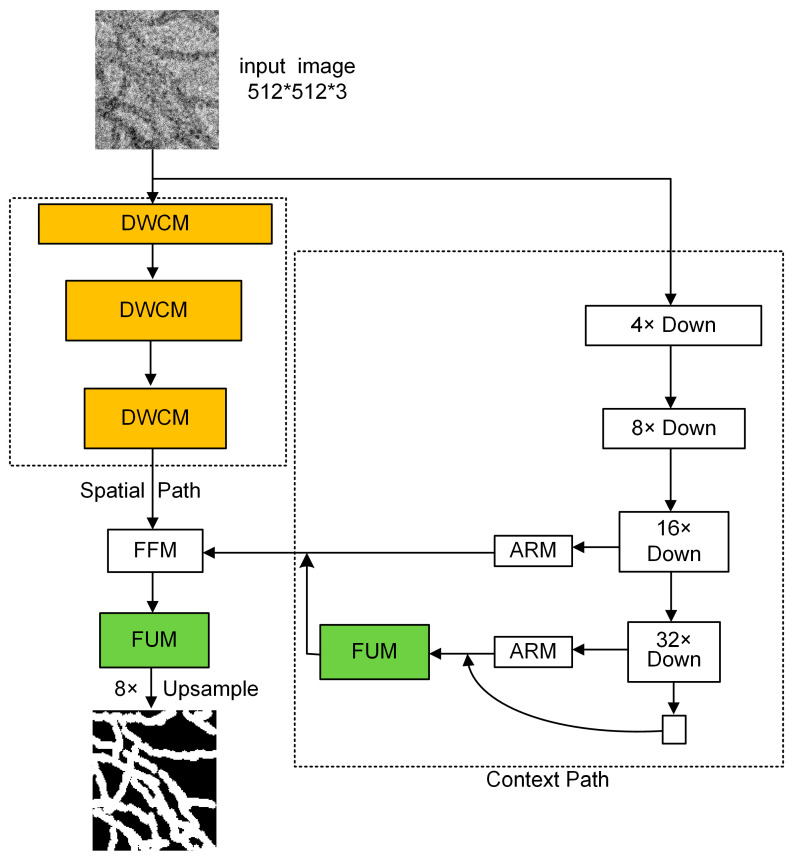
Structure of the WaveBiSeNet network.

In BiSeNetV1 [15], stride-2 convolutions in the spatial path discard high frequency edge cues and, together with the shallow depth of this path, limit the receptive field; bilinear upsampling also struggles to recover sharp boundaries. Based on BiSeNetV1 [15], we propose WaveBiSeNet, shown in Figure 3. Using a 3 × 512 × 512 image as input, the three stride-2 convolutions in the spatial path are replaced with Dual Wavelet Convolution Modules (DWCM), that perform wavelet based downsampling to separate low and high frequency subbands, explicitly retaining horizontal, vertical, and diagonal edge responses characteristic of slender one-dimensional nanowires. A second decomposition on the low frequency branch expands the effective receptive field and strengthens contextual modeling. A lightweight convolution then compresses channels. The spatial path sizes evolve as 3 × 512 × 512 → C1 × 256 × 256 → C2 × 128 × 128 → C3 × 64 × 64, with C1 = 64, C2 = 128, and C3 = 256. The context path computes features at scales of 1/4, 1/8, 1/16, and 1/32, that is 128 × 128, 64 × 64, 32 × 32, and 16 × 16, and uses attention refinement modules at 1/16 and 1/32. Immediately after the 1/32 stage we place a Flexible Upsampling Module (FUM) that predicts learnable offsets to dynamically generate sampling points and reconstruct edge details in a lightweight and efficient manner, and it upsamples that branch to 1/8 for fusion. The fused context is then combined with the 1/8 spatial features through the Feature Fusion Module to obtain Cf × 64 × 64. A second Flexible Upsampling Module replaces bilinear interpolation and performs ×8 upsampling back to 512 × 512, producing logits of size 2 × 512 × 512 for two classes. Together, DWCM edge aware downsampling and FUM shape adaptive dynamic upsampling achieve the recovery of blurred details.

#### 3.2.1. Dual Wavelet Convolution Module

**Figure 4:**
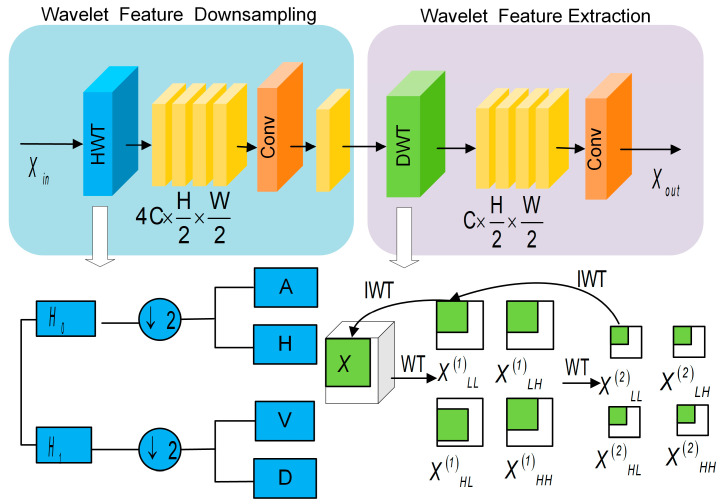
Structure of the DWCM.

Inspired by HWD [35] and WTConv [36], we propose the Dual Wavelet Convolution Module (DWCM), designed to replace the three strided convolution operations with 3 × 3 kernels in the spatial path of BiSeNetV1 [15], as shown in Figure 4. We stack three DWCMs for two main reasons. First, they one-to-one replace the three stride-2 downsampling operations in the BiSeNetV1 [15] spatial path, preserving the original downsampling schedule so that the resolution halves at each stage and ultimately aligns with the one-eighth fusion scale. Second, they enable a progressive multi-scale analysis that converts each downsampling step into a wavelet-based decomposition: the first stage prioritizes the preservation of high-frequency edges, the second consolidates mid-scale structures, and the third performs a secondary decomposition of the low-frequency component to enlarge the effective receptive field and reduce aliasing. In computer vision, convolutional filters operating in the spatial domain and Fourier transform-based filters in the frequency domain are two widely adopted methods for feature extraction. The Wavelet Transform (WT) adopted in the proposed DWCM is derived from Fourier analysis and harmonic analysis [37]. It enables the extraction of features in both the temporal and frequency domains. This dual-domain representation enhances the model’s ability to capture multi-scale information.

The DWCM consists of two main components: the Wavelet Feature Downsampling Block and the Wavelet Feature Extraction Block. The Wavelet Feature Downsampling Block applies a Haar Wavelet transform (HWT) to decompose the input feature map into four frequency subbands: low frequency, horizontal high frequency, vertical high frequency, and diagonal high frequency, which both downsample the spatial resolution and map the local structure into frequency channels, capturing edge and orientation cues of 1D nanowires. The Wavelet Feature Extraction Block further decomposes the initially obtained subband feature maps. This operation extracts richer multi-scale frequency information. The Wavelet Feature Extraction Block further decomposes the initially decomposed subband feature maps using Discrete Wavelet Transform (DWT). This operation extracts richer multi-scale frequency information. Considering that the low-frequency subband contains richer structural and contextual information, a second wavelet decomposition is performed on it. This operation enhances feature representation and further enlarges the receptive field, facilitating more accurate segmentation of one-dimensional nanowires, especially in cases with complex backgrounds and blurred edges.

Specifically, four wavelet filters are defined with a stride of 2 and a kernel size of 1 × 1. The low-frequency filter ($H_{0}$) corresponds to the low frequency convolution kernel $f_{LL}$, and is used to extract the global information of the image. The high-frequency filters ($H_{1}$) corresponding to high-frequency convolution kernels ($f_{LH}$, $f_{HL}$, and $f_{HH}$), capture detailed information of the local edges. The formulas are presented in Equation (1).

(1) $$\begin{array}{cc} {\;f}_{LL}=\left[\begin{array}{cc} 1 & 1\\ 1 & 1 \end{array}\right], & \;f_{LH}=\left[\begin{array}{cc} -1 & -1\\ 1 & 1 \end{array}\right]\\ f_{HL}=\left[\begin{array}{cc} -1 & 1\\ -1 & 1 \end{array}\right], & \;f_{LH}=\left[\begin{array}{cc} 1 & -1\\ -1 & 1 \end{array}\right] \end{array}$$

In the Wavelet Feature Downsampling Block, the nanowire feature map $X_{in}$ with dimension C×H×W first processed by the HWT. Convolution operations are performed using the low-pass filter ($H_{0}$) and the high-pass filter ($H_{1}$) in the horizontal and vertical directions, respectively. The symbol ↓ denotes the 2× downsampling operation applied to both the approximation and detail components. This operation decomposes the input into four frequency subbands: the low-frequency subband $X_{LL}$, the horizontal high-frequency subband $X_{LH}$, the vertical high-frequency subband $X_{HL}$, and the diagonal high-frequency subband $X_{HH}$. These components correspond to the approximation subband (A) and the three detail subbands: vertical (V), horizontal (H), and diagonal (D), respectively. These subbands encode spatial frequency features into separate channels, allowing for resolution reduction while preserving key edge and orientation information. The processed feature map’s size is $4C\times \frac{H}{2}\times \frac{W}{2}$. The formulas are presented in Equation (2).

(2) $$\left[X_{LL},X_{LH},X_{HL},X_{HH}\right]=\left(HWT,{\;X}_{in}\right)$$

Subsequently, a 1×1 convolution, batch normalization (BN), and ReLU activation function are applied to fuse and refine the frequency information across different channels. This operation produces a fused feature map $X^{\prime }$ with dimensions $C\times \frac{H}{2}\times \frac{W}{2}$; this operation effectively integrates multi-frequency information. It enhances the model’s ability to perceive both local details and global structures. As a result, the segmentation accuracy is improved. The corresponding formula is presented in (3).

(3) $$X^{\prime }=ReLU\left(BN\left({Conv}_{1\times 1}\left(Concat\left(X_{LL},\;X_{LH},{\;X}_{HL},{\;X}_{HH}\right)\right)\right)\right)$$

In the Wavelet Feature Extraction Block, the downsampled feature map $X^{\prime }$ is further processed using the WT. Through this operation, it is decomposed into four subbands: the low-frequency component $X_{LL}^{\left(1\right)}$, the horizontal high-frequency component $X_{LH}^{\left(1\right)}$, the vertical high-frequency component $X_{HL}^{\left(1\right)}$, and the diagonal high-frequency component $X_{HH}^{\left(1\right)}$, as shown in Equation (4).

(4) $$\left[X_{LL}^{\left(1\right)},X_{LH}^{\left(1\right)},\;X_{HL}^{\left(1\right)}\;,X_{HH}^{\left(1\right)}\right]={Conv}_{1\times 1}\left(WT,{\;X}^{\prime }\right)$$

Because the low-frequency component frequently contains richer and finer-grained frequency information, the low-frequency information $X_{LL}^{\left(1\right)}$ is decomposed into low-frequency information by another WT, yielding four subbands $X_{LL}^{\left(2\right)}$, $X_{LH}^{\left(2\right)}$, $X_{HL}^{\left(2\right)}$, and $X_{HH}^{\left(2\right)}$. The formula is shown in Equation (5).

(5) $$\left[X_{LL}^{\left(2\right)},X_{LH}^{\left(2\right)},\;X_{HL}^{\left(2\right)}\;,X_{HH}^{\left(2\right)}\right]={Conv}_{1\times 1}\left(WT,\;\;X_{LL}^{\left(1\right)}\right)$$

Multilevel feature maps at different frequency scales are finally obtained. The low-frequency component $Y_{LL}^{\left(1\right)}$, and the high-frequency component $Y_{H}^{\left(1\right)}$, are generated by fusing the first-level wavelet decomposition results using a 1×1 convolution. Similarly, the second-level decomposition results are fused to produce $Y_{LL}^{\left(2\right)}$ and ${\;Y}_{H}^{\left(2\right)}$. This fusion enhances the low-frequency feature representation, which improves the model’s ability to capture contextual information, as shown in Equation (6).

(6) $$\begin{array}{c} Y_{LL}^{\left(1\right)},Y_{H}^{\left(1\right)}={Conv}_{1\times 1}\left(W^{\left(1\right)},\left(X_{LL}^{\left(2\right)},{\;X}_{LH}^{\left(2\right)},{\;X}_{HL}^{\left(2\right)},{\;X}_{HH}^{\left(2\right)}\right)\right)\\ Y_{LL}^{\left(2\right)},Y_{H}^{\left(2\right)}={Conv}_{1\times 1}\left(W^{\left(2\right)},\left(X_{LL}^{\left(1\right)},{\;X}_{LH}^{\left(1\right)},\;X_{HL}^{\left(1\right)},{\;X}_{HH}^{\left(1\right)}\right)\right) \end{array}$$

The Inverse Wavelet Transform (IWT) was applied to recombine information from different frequency bands, producing an intermediate feature map. Then, a 1×1 convolution was employed to adjust the number of output channels. This process yields the final feature map ${\;X}_{out}$ with dimensions $4C\times \frac{H}{2}\times \frac{W}{2}$, as described in Equation (7).

(7) $$X_{out}={Conv}_{1\times 1}\left(IWT\left(Y_{LL}^{\left(1\right)}+Y_{LL}^{\left(2\right)},\;Y_{H}^{\left(1\right)}+Y_{H}^{\left(2\right)}\right)\right)$$

#### 3.2.2. Flexible Upsampling Module

**Figure 5:**
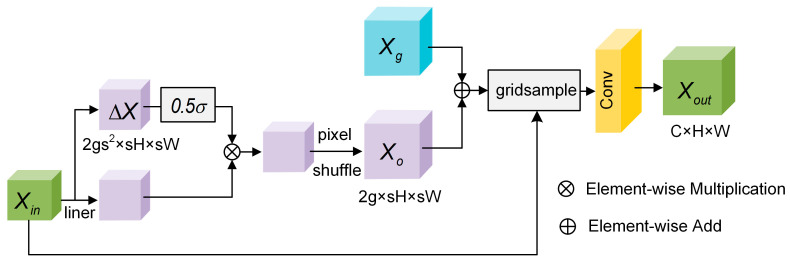
Structure of the FUM.

Inspired by DySample [38], we propose a lightweight, flexible upsampling module, the Flexible Upsampling Module (FUM). In this module, a convolution layer is added after the DySample [38] grid sampling operation to better capture and refine the features of one-dimensional nanowires. Its architecture is illustrated in Figure 5. By introducing learnable offsets, sampling positions are dynamically generated. This design enables the efficient reconstruction of fine-grained features of one-dimensional nanowires and the recovery of blurred details, while maintaining low computational cost.

Specifically, the input feature map $X_{in}\in R^{C\times H\times W}$ is first used to construct a standard sampling grid $X_{g}\in R^{2\times H\times W}$, where each sampling position is represented by two coordinate values. Subsequently, $X_{in}$ is linearly transformed to produce an offset feature map $∆X\in R^{{2gs}^{2}\times H\times W}$. Here, g denotes the number of channel groups, s represents the upsampling scale factor, and the factor 2 indicates the horizontal and vertical offsets for each coordinate. To constrain the range of the offsets, the offset map is normalized to the interval $\left[0,1\right]$ using a sigmoid function. It is then multiplied by a learnable dynamic scaling factor 0.5, which is adapted based on the image features, to further adjust the sampling positions. The result is element-wise multiplied with the input feature map $X_{in}$, Then, the channel information is remapped to the spatial domain using a pixel_shuffle operation with an upsampling scale factor r, producing the sampling ensemble network $X_{o}\in R^{2g\times H\times W}$. The final sampling grid $X_{S}$ is obtained by summing $X_{g}$ and $X_{o}$. Based on the coordinates of each pixel in $X_{S}$, bilinear interpolation is performed on $X_{in}$ using the grid_sample function. The sampled feature maps are sequentially passed through a 1×1 convolution layer, followed by Batch Normalization (BN) and a ReLU activation function. This process fuses channel information and introduces nonlinear transformations, which help to mitigate the internal covariate shift. As a result, the reconstruction of the 1D nanowire edges is improved. The output feature map $X_{out}\in R^{C\times (H\cdot r)\times (W\cdot r)}$ is obtained. The calculation process is shown in Equation (8).

(8) $$\left\{\begin{array}{c} X_{in\;}\in R^{C\times H\times W}\\ X_{o}=\mathrm{pixel}\_\mathrm{shuffle}(\left(0.5\sigma \left(w\cdot X_{in}\right)\right)\cdot \left(w\cdot X_{in}\right))\\ X_{s\;}=X_{g\;\;}+X_{o}\\ X_{out\;}=\mathrm{ReLU}(\mathrm{BN}({\mathrm{Conv}}_{1\times 1}(\mathrm{grid}\_\mathrm{sample}(X_{in},X_{s})))) \end{array}\right.$$

## 4. Results and Discussion

### 4.1. Dataset

**Figure 6:**
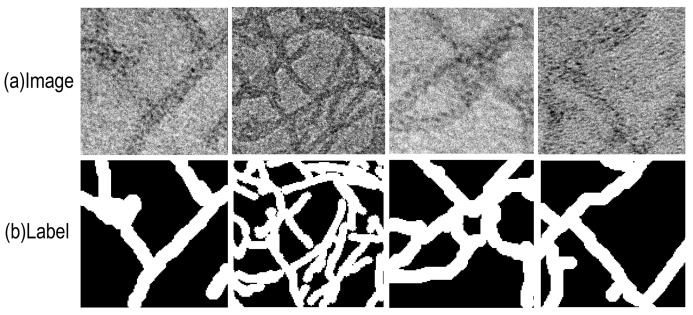
Peptide nanowire dataset. (**a**) original image; (**b**) label image.

This study utilized a dataset of Transmission Electron Microscopy (TEM) images of peptide nanowires, synthesized and imaged by Brian Montz in Professor Todd Emrick’s research group at the Department of Polymer Science and Engineering, University of Massachusetts Amherst [39], and made available through the open-access repository Zenodo [40]. The dataset comprised two categories: dispersed and network structures, with 100 images each. To accurately delineate nanowire regions, we first applied a 3 × 3 median filter to the original grayscale TEM images for denoising. The denoised images were then converted to the Hue–Saturation–Value (HSV) color space, and dual thresholds S < 40 and V < 90 were used to obtain initial binary masks. We performed one opening operation with a 3 × 3 structuring element to remove isolated noise, followed by two closing operations to repair small breaks; finally, we dilated the foreground by one pixel to better approximate the physical diameter. To address common issues in thin nanowires—segmented breaks and edge under-segmentation—we adopted SAM-assisted manual correction based on the Segment Anything Model [41]. Annotators used interactive prompts to generate candidate masks and performed pixel-level refinements, after which the results were reviewed and accepted as the final ground truth. In the label maps, nanowire regions are shown in white and the background in black. As shown in Figure 6, “Image” denotes the original TEM image and “Label” the corresponding binary label map. We first split the dataset at the original-image level into training, validation, and test sets in a 7:2:1 ratio, and then cropped each original image into fixed-size patches, yielding a total of 3035 samples: 2135 for training, 600 for validation, and 300 for testing.

### 4.2. Experimental Setup

The model was trained on a server equipped with an NVIDIA GeForce RTX 4090 (24 GB) GPU. The training environment included Python 3.9.19, PyTorch 1.8.1, and CUDA 12.4. Prior to training, the dataset was normalized to [0, 1], and He initialization was applied to optimize network weights and accelerate convergence. To improve the model’s sensitivity to 1D nanowire boundaries, a combined loss function was employed, consisting of 0.5 times the cross-entropy loss [42] and 0.5 times the weighted Dice loss [43]. Parameters were updated using the Adam optimizer [44], with an initial learning rate of 0.01 and momentum parameters β_1_ and β_2_ both set to 0.99. L2 regularization with a weight decay of 0.001 was applied to prevent overfitting. The learning rate was dynamically adjusted via the ReduceLROnPlateau scheduler. When the monitored metric plateaued, the learning rate was reduced by a factor of 0.5. The batch size was set to 16. The model was trained for 300 epochs.

### 4.3. Evaluation Metric

To quantitatively evaluate the segmentation performance of the WaveBiSeNet model on 1D nanowire materials, we selected four evaluation metrics in our experiments to comprehensively reflect the model’s performance from different aspects: mean intersection over union(mIoU) [45,46,47], accuracy [48,49], F1 score [50,51], and Kappa coefficient (Kappa) [52].

mIoU [45,46,47] is a widely used metric for evaluating image segmentation accuracy. It quantifies the pixel-level overlap between predicted segmentation and ground-truth annotations. The mIoU is computed as the average Intersection over Union (IoU) across all classes, and its formulation is provided in Equation (9).

(9) $$mIoU=\frac{1}{n_{c}}\sum_{i}\frac{n_{ii}}{\sum_{i}n_{ij}+\sum_{j}n_{ji}-n_{ii}}$$

In the confusion matrix, $n_{ij}$ denotes the number of pixels that belong to class i but are incorrectly predicted as class j. Similarly, $n_{ji}$ represents the number of pixels that belong to class j but are misclassified as class i. The value $n_{ii}$ represents the number of pixels correctly predicted as class i. The variable $n_{c}$ refers to the total number of classes in the image.

Accuracy [48,49] is a fundamental metric for measuring the performance of a classification model, as it represents the proportion of correctly classified samples among the total samples. The formula is shown in Equation (10).

(10) $$Accuracy=\frac{TP+TN}{TP+TN+FP+FN}$$

Here, TP refers to the number of foreground pixels correctly classified as foreground. TN denotes background pixels correctly classified as background. FP represents background pixels incorrectly classified as foreground, while FN indicates foreground pixels misclassified as background.

We present the confusion matrix for the improved model:


**Prediction**
ReferenceTP: 19,502,345FN: 1,636,842FP: 5,798,124TN: 51,705,679

F1 score [50,51] is a composite metric that balances precision [53,54] and recall [53,54], providing a more comprehensive assessment of the model’s ability to detect foreground regions. The formula is shown in Equation (11).

(11) $$F1\;score=2\times \frac{Precision\times Recall}{Precision+Recal}$$

The Kappa coefficient [52] is used to assess the classification consistency of the model in segmentation tasks. It quantifies the improvement of the segmentation results over those expected from random classification. In addition, it effectively corrects for the influence of class imbalance on performance evaluation. The coefficient reflects the actual classification performance by comparing the observed agreement $p_{o}$ with the expected agreement by chance $p_{e}$. The calculation formula is provided in Equation (12).

(12) $$Kappa=\frac{p_{o}-p_{e}}{1-p_{e}}$$

where $p_{o}$ denotes the actual proportion of all pixels correctly classified by the model. It refers to the ratio of correctly predicted foreground and background pixels to the total number of pixels. In contrast, $p_{e}$ denotes the expected proportion of correctly classified pixels under random category assignment. It is estimated based on the prior distribution of foreground and background classes.

### 4.4. Ablation Experiments

To validate the effectiveness of the proposed modules, ablation experiments were conducted on the peptide nanowire dataset. The experimental results are presented in Table 1. The Dual Wavelet Convolution Module (DWCM) preserved edge details while reducing spatial resolution through efficient downsampling and receptive field expansion. This resulted in a model mIoU of 77.29%. The Flexible Upsampling Module (FUM) also demonstrated good performance in recovering fine-grained features, achieving an mIoU of 76.32%. When both modules were applied simultaneously, the mIoU increased to 77.59%. Compared with the baseline model BiSeNetV1 [15], the proposed modules led to improvements in mIoU, accuracy, F1 score, and Kappa. These results indicate the effectiveness of the proposed design in the 1D nanowire segmentation task.

**Figure 7:**
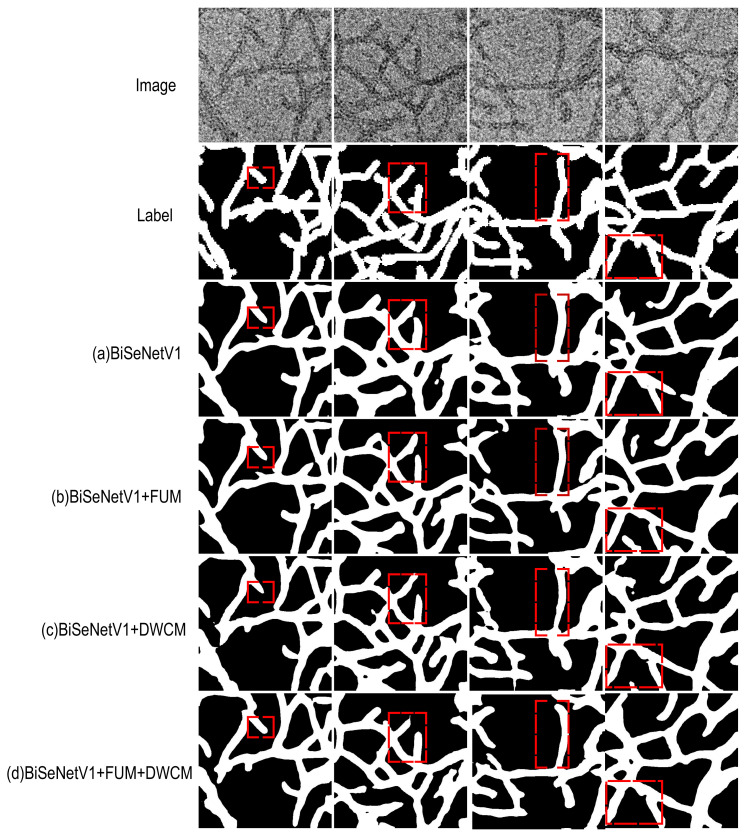
Model ablation experiment results. (**a**) BiSeNetV1; (**b**) BiSeNetV1+FUM; (**c**) BiSeNetV1 + DWCM; (**d**) BiSeNetV1 + FUM + DWCM.

We conducted visual ablation experiments to evaluate the effectiveness of the proposed modules. The results are presented in Figure 7. This figure illustrates the segmentation performance of the BiSeNetV1 [15] semantic segmentation model with different module combinations. The “Image” refers to the original input, and the “Label” denotes the corresponding ground truth. Key segmentation regions are highlighted with red dashed boxes for comparison. Compared to the baseline BiSeNetV1 [15], the DWCM improves edge detection and enhances the recognition of local structures. The FUM significantly improves detail recovery, resulting in smoother boundaries. When both modules are integrated, the model achieves the best performance in segmenting nanowire edges. Structural adhesion is effectively reduced, and boundary separation becomes clearer.

### 4.5. Model Comparison and Analysis

To comprehensively evaluate the segmentation performance of WaveBiSeNet, comparative experiments were conducted using the same allocation ratio of the peptide nanowire dataset. We used different random seeds to train each model five times and reported the accuracy results as the mean ± standard deviation. The results were compared with a series of state-of-the-art real-time segmentation algorithms, including BiSeNetV1 [15], FSSNet [55], EDA-Net [56], ESNet [57], CANet [58], AGLNet [59], BiSeNetV2 [60], Mobile-Unet [61], EGE-Unet [57], and LETNet [62]. As shown in Table 2, WaveBiSeNet achieved competitive performance across multiple evaluation metrics when ResNet18 [34] was used as the backbone network. Specifically, it achieved an mIoU of 77.59%, an accuracy of 89.95%, an F1 score of 87.22%, and a Kappa coefficient of 74.13%. Compared with the baseline model BiSeNetV1 [15], WaveBiSeNet improved these metrics by 2.12%, 1.26%, 1.56%, and 2.62%, respectively. EGE-Unet [57] attains 75.84% mIoU, which is slightly lower than WaveBiSeNet. FSSNet [55]; EDA-Net [56], ESNet [57], and CANet [58] achieved mIoUs between 68% and 72%, making them more suitable for resource-constrained scenarios. BiSeNetV2 [60] achieved an mIoU of 65.50%, offering a lightweight and efficient solution but with slightly reduced precision. AGLNet [59], LETNet [62], and Mobile-Unet [61] reported mIoUs between 60% and 66%, with most Kappa scores below 80%. These models exhibited limited capability in capturing fine details and delineating boundaries, which makes the segmentation of nanowire images particularly challenging, especially when the precise recognition of tiny structures is needed. Overall, WaveBiSeNet effectively preserves fine details while capturing broader contextual information, improving the segmentation of one-dimensional nanowires.

**Figure 8:**
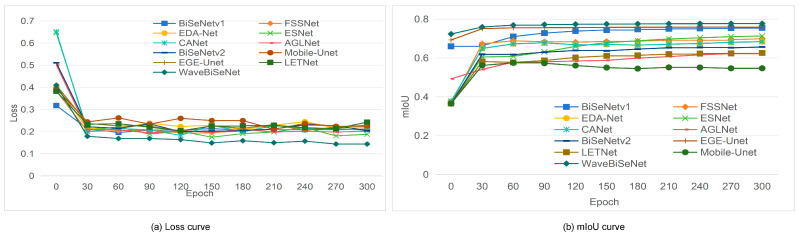
Curves of different semantic segmentation networks. (**a**) Loss curve; (**b**) mIoU curve.

To further evaluate model performance, the Loss and mIoU curves over 300 training epochs are plotted in Figure 8. The Loss curve is shown in Figure 8a, and the mIoU curve is shown in Figure 8b.

As shown in Figure 8a, all models exhibit rapid loss reduction within the first 30 epochs, followed by convergence. ESNet [57] and CANet [58] start with the highest losses (>0.6) and show unstable training. BiSeNetV1 [15], BiSeNetV2 [60], and AGLNet [59] converge more quickly to 0.18, with moderate oscillations. Mobile-Unet [61] and EGE-Unet [57] remain less stable, ending between 0.20 and 0.25, while FSSNet [55] and EDA-Net [56] converge slowly around 0.22. LETNet [62] achieves a stable loss of 0.15 but converges slower than WaveBiSeNet. In contrast, WaveBiSeNet converges faster, stabilizing at 0.13 after epoch 210, which reflects faster convergence, lower final loss, and improved training stability.

From Figure 8b, all models show rising mIoU in the first 30 epochs before stabilization. BiSeNetV1 [15], BiSeNetV2 [60], EGE-Unet [57], and AGLNet [59] reach 0.73–0.75, but fluctuate in later stages. ESNet [56], CANet [57], and EDA-Net [56] remain limited (0.66–0.69), while Mobile-Unet [61] and LETNet [62] perform the worst (0.55–0.60), with Mobile-Unet [61] converging prematurely. FSSNet [55] stabilized at 0.69. Compared with other models, WaveBiSeNet achieves faster convergence (within 60 epochs) and a higher final mIoU of 0.77. These results suggest that the proposed improvements enhance its effectiveness for one-dimensional nanowire segmentation, particularly under challenging background conditions.

**Figure 9:**
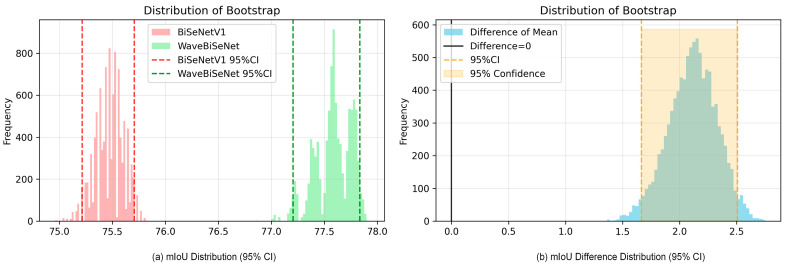
Bootstrap analysis of mIoU between BiSeNetV1 and WaveBiSeNet. (**a**) mIoU distributions with 95% confidence intervals; (**b**) mIoU difference distribution with 95% confidence intervals.

We conducted a statistical significance analysis of BiSeNetV1 and WaveBiSeNet using the bootstrap method with 10,000 resampling iterations, and the results are presented in Figure 9. Figure 9a illustrates the mIoU distributions of the two models. The histogram of BiSeNetV1, shown in red, peaks at approximately 75.5%, indicating that most of its mIoU values are concentrated at this level. The distribution of WaveBiSeNet, shown in green, peaks at around 77.5% and is clearly shifted to the right, reflecting better overall performance. The 95% confidence intervals, marked by dashed lines, are approximately 75.0–75.8% for BiSeNetV1 and 77.0–78.0% for WaveBiSeNet. Since these intervals do not overlap, the results provide an initial indication that WaveBiSeNet achieves significantly higher mIoU than BiSeNetV1. Figure 9b shows the distribution of the mIoU differences between the two models. The light blue histogram represents the differences obtained after each bootstrap resampling. The black solid line indicates the baseline of no difference (Difference = 0), while the orange dashed lines and shaded area represent the 95% confidence interval of the difference, approximately ranging from 1.5 to 2.5. This interval lies entirely to the right of zero and does not intersect the baseline, demonstrating that the observed improvement is not due to random variation but is statistically significant at the 95% confidence level.

In summary, the bootstrap analysis confirms that WaveBiSeNet achieves a significantly higher mIoU than BiSeNetV1. This performance improvement is statistically significant (*p* < 0.05), supported by the non-overlapping confidence intervals and the strictly positive difference distribution.

### 4.6. Visual Comparison of Segmentation Performance

**Figure 10:**
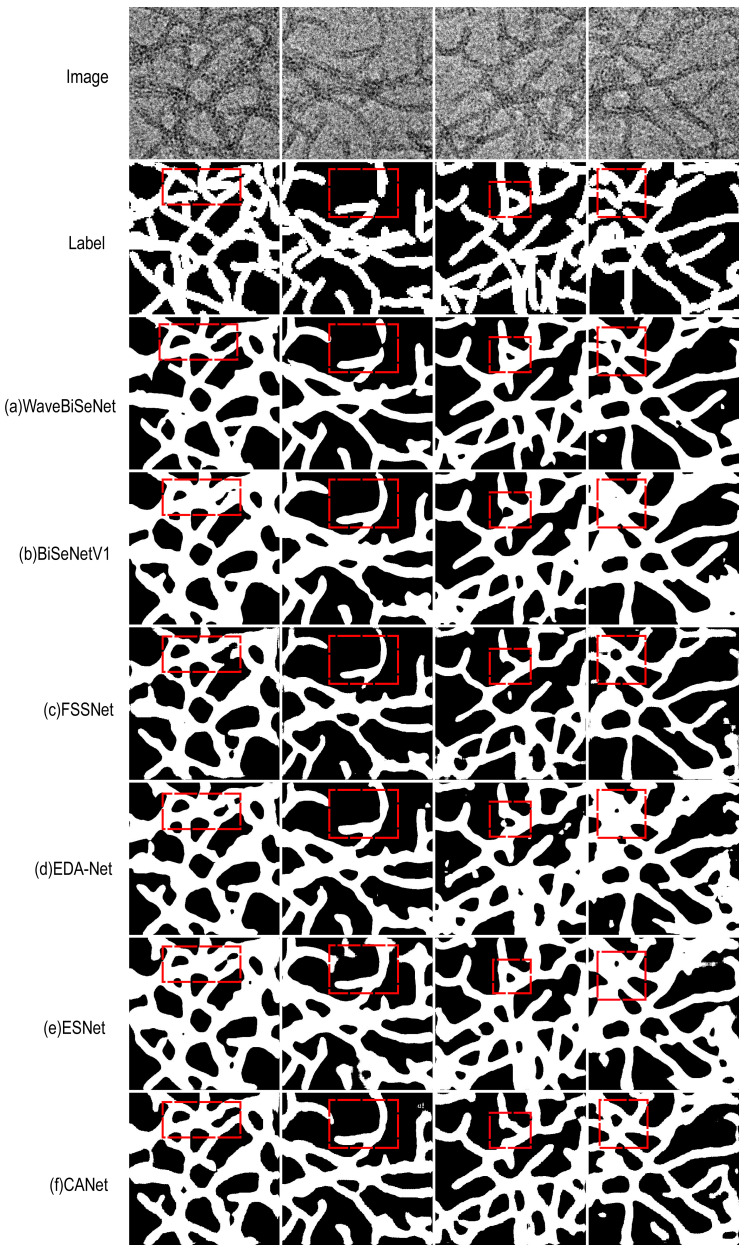

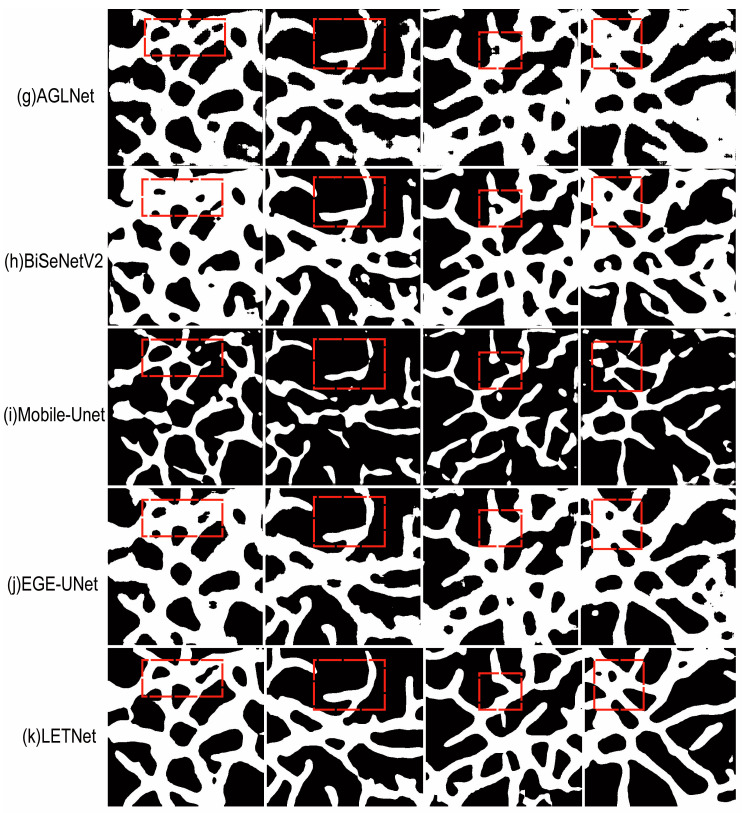
Model prediction results. (**a**) WaveBiSeNet; (**b**) BiSeNetV1; (**c**) FSSNet; (**d**) EDA-Net; (**e**) ESNet; (**f**) CANet; (**g**) AGLNet; (**h**) BiSeNetV2; (**i**) Mobile-Unet; (**j**) EGE-Unet; (**k**) LETNet.

We randomly selected four images from the test set to visualize and compare the prediction results of each model, as shown in Figure 10. From top to bottom, the first row presents the original images (Image), the second row shows the ground truth labels (Label), the third row displays the predictions of the proposed WaveBiSeNet, and the subsequent rows correspond to the predictions of other compared models. Key segmentation regions are highlighted with red dashed boxes to illustrate performance differences among models. In the dispersed state, 1D nanowires show slight overlap, while in complex states, extensive interweaving and background blurring are observed. Such structural complexity often leads to inaccuracies and adhesion in edge segmentation. The visualization results indicate that WaveBiSeNet provides clearer edge restoration, with an improved distinction of fine structures and reduced adhesion between regions. This model excels in preserving local features, such as elongated linear structures and branches. In contrast, BiSeNetV1 [15], FSSNet [55], CANet [58], and LETNet [62] segment large regions reasonably well but are less accurate at fine edges. EDA-Net [56], ESNet [57], AGLNet [59], BiSeNetV2 [60], and EGE-Unet [57] exhibit edge adhesion problems, indicating limited ability to handle complex boundaries. Mobile-Unet [61] performs the worst, with evident segmentation errors and detail omissions, failing to capture complex structures accurately. In summary, WaveBiSeNet demonstrates robust performance in segmentation accuracy, edge restoration, and fine-detail recognition for 1D nanowire images.

## 5. Conclusions

To address the key challenges in one-dimensional nanowire segmentation, including blurred backgrounds, indistinct edges, and low image contrast, we propose a wavelet-based bilateral segmentation network (WaveBiSeNet). The model was trained and validated on a peptide nanowire dataset. Experimental results show that WaveBiSeNet achieves a mIoU of 77.59%, an Accuracy of 89.95%, an F1 score of 87.22%, and a Kappa coefficient of 74.13%. Under the same data split and evaluation protocol with ResNet18 as the backbone, WaveBiSeNet improves over BiSeNetV1 by 2.12 percentage points in mIoU, 1.26 percentage points in accuracy, 1.56 percentage points in F1 score, and 2.62 percentage points in Kappa. Qualitative analyses indicate that the method effectively handles adherent regions in one-dimensional nanowire materials and exhibits strong robustness under ambiguous backgrounds. Built upon the BiSeNetV1 architecture, the network introduces a Dual Wavelet Convolution Module and a Flexible Upsampling Module. While reducing spatial resolution, it maximally preserves structural information at the edges of the nanowires and enhances context awareness, thereby recovering blurred edge details more faithfully. Overall, WaveBiSeNet provides an accurate and efficient solution for 1D nanowire segmentation. Future work will focus on further optimizing the network architecture to improve segmentation performance and adaptability. Efforts will also be made to enhance the model’s compatibility with various nanomaterial types. Additionally, cross-modal transfer learning and few-shot learning strategies will be explored to facilitate the practical application of deep learning-based semantic segmentation in nanomaterials research.

## Figures and Tables

**Table 1:** Comparison of ablation experiments on the peptide nanowire dataset.

Method	mIoU (%)	Accuracy (%)	F1 (%)	Kappa (%)
BiSeNetV1	75.47% ± 0.31	88.69% ± 0.26	85.66% ± 0.40	71.51% ± 0.62
BiSeNetV1 + FUM	76.32% ± 0.35	89.34% ± 0.24	86.44% ± 0.34	72.40% ± 0.46
BiSeNetV1 + DWCM	77.29% ± 0.38	89.75% ± 0.25	86.73% ± 0.35	76.62% ± 0.49
BiSeNetV1 + FUM + DWCM	77.59% ± 0.42	89.95% ± 0.23	87.22% ± 0.30	74.13% ± 0.33

**Table 2:** Network model test results.

Models	Backbone	MIoU (%)	Accuracy (%)	F1 (%)	Kappa (%)
WaveBiSeNet	ResNet18	77.59% ± 0.42	89.95% ± 0.23	87.22% ± 0.30	74.13% ± 0.33
BiSeNetV1	ResNet18	75.47% ± 0.31	88.69% ± 0.26	85.66% ± 0.40	71.51% ± 0.62
FSSNet	-	69.53% ± 0.69	85.22% ± 0.55	81.47% ± 0.60	63.43% ± 0.53
EDA-Net	-	68.18% ± 0.66	84.51% ± 0.45	80.45% ± 0.69	61.14% ± 0.64
ESNet	-	71.21% ± 0.31	86.13% ± 0.53	82.48% ± 0.55	65.34% ± 0.63
CANet	MobilenetV2	71.28% ± 0.62	86.57% ± 0.67	82.81% ± 0.60	65.63% ± 0.51
AGLNet	-	62.50% ± 0.45	79.95% ± 0.56	75.76% ± 0.58	52.13% ± 0.43
BiSeNetV2	-	65.50% ± 0.35	82.46% ± 0.34	78.31% ± 0.40	57.21% ± 0.35
Mobile-Unet	MobileNet	54.37% ± 0.46	75.22% ± 0.58	69.51% ± 0.30	38.64% ± 0.57
EGE-Unet	-	75.84% ± 0.40	88.91% ± 0.62	85.84% ± 0.46	71.75% ± 0.38
LETNet	-	62.55% ± 0.35	80.44% ± 0.35	76.01% ± 0.35	52.20% ± 0.45

## Data Availability

The raw data supporting the conclusions of this article will be made available by the authors on request.
